# A bifactor model of personality organization in adolescence: the validity of a brief screening measure assessing severity and core domains of functioning

**DOI:** 10.1186/s12888-022-03926-y

**Published:** 2022-07-08

**Authors:** M. Biberdzic, B. F. Grenyer, L. Normandin, K. Ensink, J. F. Clarkin

**Affiliations:** 1 Illawarra Health and Medical Research Institute and School of Psychology, University of Wollongong, Wollongong, Australia; 2 Department of Psychology, Laval University, Quebec, QC Canada; 3 Personality Disorders Institute and Department of Psychiatry, Weill Medical College of Cornell University, New York, NY USA

**Keywords:** Personality disorder, Adolescents, Bifactor model, Personality organisation, Personality functioning, Severity

## Abstract

**Background:**

Both the latest edition of the DSM-5 as well as the new ICD-11 have established a new focus in the diagnosis of personality disorders: the assessment of personality functioning. This recent shift in focus converges with long-standing psychodynamic conceptualizations of personality pathology, particularly Kernberg’s object relations model. Although a significant amount of research supports these models in adults, much less is known about the validity of these frameworks in youth. Considering the paucity of brief measures of personality functioning in adolescents, the current study aimed to develop and investigate the validity of the Inventory of Personality Organization for Adolescents—Short Form, a theoretically-informed measure assessing severity and core domains of functioning in adolescents.

**Methods:**

A total sample of *N* = 525 adolescents aged 13 to 19 years were recruited through a community University-Health Psychology Clinic as current patients (*n* = 94) or who responded to an online research call (*n* = 431).

**Results:**

Results indicate that a bifactor model provided the best fit to the data and consisted of a general factor reflecting core self-other functioning and three specific factors, representing additional dimensions of personality organization.

**Conclusions:**

A brief 15-item version of the IPO-A was successfully derived for time-efficient screening of personality pathology in youth. Similarities with the ICD-11 framework are discussed.

**Supplementary Information:**

The online version contains supplementary material available at 10.1186/s12888-022-03926-y.

## Background

In light of the well-documented shortcomings of the *DSM*’s categorical conceptualization of personality disorders (PDs), alternative diagnostic frameworks for understanding and classifying personality pathology have been developed. Both the latest editions of the Diagnostic and Statistical Manual of Mental Disorders (DSM-5) and the International Classification of Diseases (ICD-11) have established a new focus in the diagnosis of PDs: the assessment of *personality functioning*. In these dimensional models, impairment in self and interpersonal function represents a general diagnostic criterion for any PD, an understanding that converges with long-standing psychodynamic conceptualizations of personality pathology [1]. More specifically, a close association can be found between the conceptualization of the DSM-5’s Level of Personality Functioning Scale (LPFS) and the concept of *personality organization* as defined in object relations theory [1]. Although a significant amount of research supports the use of these new diagnostic frameworks in adults [2], much less is known about their clinical utility in youth [3]. Moreover, despite growing consensus in the field regarding the importance of early intervention, there still remains a dearth of valid and time effective measures for assessing maladaptive personality functioning in adolescents [4]. Considering the current lack of brief measures of personality (dys)function in youth, and the recent development of a dimensional measure of personality organization in adolescents (the Inventory of Personality Organization for Adolescents; IPO-A [5]), the main objective of the current study was to develop and validate a shorter version of the IPO-A, and investigate its clinical utility in screening for impairments in personality functioning compared to the DSM-5’s LPFS.

### The DSM-5 alternative model of personality disorders

Despite ultimately retaining a categorical model for the assessment of PDs, the DSM-5 included under Section III its dimensional system, the Alternative Model for Personality Disorders (AMPD). The AMPD conceptualises personality dysfunction along a continuum of severity that includes impairments in both self (identity and self-direction) and interpersonal (empathy and intimacy) domains [6]. Impairment across these domains is defined as the core feature of any PD (Criterion A). In addition, a set of 25 pathological personality traits that constitute five distinct trait domains (detachment, antagonism, negative affectivity, disinhibition, psychoticism) may be used to provide more specific information regarding the personality type (Criterion B; [7]). Specifically, Criterion A is operationalized through the *Level of Personality Functioning Scale* (LPFS;[8, 9]) and Criterion B with the *Personality Inventory for DSM-5* (PID-5;[7]).

The AMPD therefore offers a model of personality pathology that aims to integrate two divergent literatures, a literature underlining self–other functioning based on object relations [10, 11] and interpersonal theory [12, 13], and another focusing on the five-factor model of personality traits [14]. It also requires the use of two different measures to assess severity of personality functioning and pathological personality traits. Moreover, as highlighted by Sexton and colleagues [15], many authors have moved away from the initial multidimensional conceptualisation of personality functioning, which is now largely operationalized as a single dimension of “severity” [16] even though research suggests its domains have descriptive value as they are not equally reflective of severity of personality pathology [15, 17]. For example, impairments in empathy in a patient with borderline PD are more likely to be associated with greater personality pathology than, for example, impairments in self-direction in another patient with the same diagnosis [18]. Nonetheless, the AMPD has been primarily used for describing PDs along 5 maladaptive personality trait dimensions, plus a single dimension of severity. This has raised concerns that the operationalization of self and interpersonal functioning may be oversimplified, while also providing little information on how to clinically integrate deficits in personality functioning with said maladaptive personality traits [18–20]. Alternatively, it has been suggested [20] that a more comprehensive and theoretically informed model such as the one elaborated by Kernberg and colleagues [11, 21] may allow for a more cohesive and clinically useful assessment of personality dysfunction.

### Kernberg’s model of personality functioning (organization)

Over the years, Kernberg and colleagues [11, 21, 22] have elaborated a model of personality functioning that revolves around the concept of personality organization (PO). PO refers to “a set of enduring, mostly unconscious psychological structures that dynamically organizes mental processes and contents into a coherent organization” ([23, 24], p.356). In recent years, the concept of PO has become a fundamental notion in contemporary psychoanalytic approaches to both adaptive and maladaptive personality [25–27], and is assumed to play a central role in the development of PDs [26, 28] and their treatment [21].

Recent papers comparing Kernberg’s object relations theory and the AMPD and ICD-11 frameworks have highlighted the broader definition of personality functioning found in psychodynamic models and the inclusion of additional dimensions necessary for the accurate assessment of one’s level of functioning [20, 29, 30]. For example, Defense mechanisms, ranging from higher functioning (e.g. repression) to more extreme processes (e.g. splitting) are not included in the AMPD but are assessed in Kernberg’s model. Also, Moral functioning, which can vary from rigid adherence to rules and excessive guilt to an absence of internalized values (as found, for example, in antisocial personality disorder), is an important indicator of severity in this model. Similarly, both Reality testing and Aggression towards self and others are considered primary determinants of personality functioning and of severity of personality pathology [21] in object relations theory whereas in the AMPD these features are considered optional “trait specifiers” [20].

Kernberg was also the first to define different levels of personality functioning and to propose key dimensions and processes involved in the development of both adaptive and maladaptive personality functioning [26]. According to Kernberg, individuals with normal (and neurotic) personality functioning show a consolidated identity, good quality of object relations, mature defence mechanisms, integrated moral functioning, preserved reality testing, and are able to control their aggressive impulses. Individuals with impaired personality functioning suffer from identity diffusion, i.e., their internal representations of self and significant others are contradictory, superficial, and not integrated. Moreover, they are not able to maintain stable interpersonal relationships, use predominantly primitive defence mechanisms to cope with stress, suffer from impaired impulse control, especially in terms of self-directed and other-directed aggression, and tend to present with impaired moral functioning. The most severely disturbed patients function on a psychotic level, accompanied by greater impairment on the previously mentioned domains of personality functioning while also presenting with impaired reality testing. Therefore, in this model, the core dimensions of PO don’t carry the same diagnostic “weight” and are associated with different clinical presentations. Although the model emphasizes varying levels of PO along a severity dimension, PO cannot be reduced to a simple scale of severity.

The validity and clinical utility of Kernberg's model of PO has been empirically supported in both clinical and non-clinical adult populations [31–37], with the concept of PO being used to assess structural components of personality functioning [25, 38]. Despite the paucity of similar work among adolescent populations, the importance of assessing PO in youth has recently been supported [5, 39] and is in line with the recent call for the development and validation of brief and developmentally sensitive measures of personality functioning in youth [3, 40].

### Maladaptive personality functioning in youth

A growing body of research suggests that diagnostic features specific to personality disorders are sufficiently stable in adolescence [41–43] and that all PD types can also be identified in youth [43–45]. Clinicians and researchers, in support of an early diagnosis of PD, argue that early detection and early intervention are crucial to prevent severe impairment of the developing personality. However, despite a renewed enthusiasm for the assessment of adolescent personality pathology in recent years, empirical support for measures of adolescent personality functioning remains scarce. Also, measures used to assess adolescent functioning either take a substantial amount of time to administer, or when brief, are narrow in scope. To date, only one self-report measure building on the LPFS has been developed, namely the Levels of Personality Functioning Questionnaire for adolescents (LoPF-Q 12–18;[3]). Although the authors report promising psychometric results, the LoPF-Q remains a lengthy instrument to use with adolescents. It also remains unclear whether a broader conceptualisation of personality functioning that includes core developmental features such as aggression modulation and moral functioning, would be more clinically useful to include in the assessment of adolescent personality pathology. In line with Kernberg’s structural theory of PO, the IPO-A may be a promising candidate.

### The Inventory of Personality Organization for Adolescents (IPO-A)

Since the introduction of the *Structural Interview* [46], a variety of instruments for the assessment of PO have been developed. Along with the Structured Interview of Personality Organization (STIPO; (Clarkin JF, Caligor E, Stern B, Kernberg OF: Structured interview of personality organization (STIPO), unpublished)), Clarkin and colleagues have developed the Inventory of Personality Organization (IPO;[47]), a self-report instrument that has demonstrated sound psychometric properties in both clinical and non-clinical adult populations (for a review, see [31]). More recently, an adapted version of the IPO has been developed for adolescents (IPO-A; (Biberdzic M, Ensink K, Normandin L, Clarkin J, Kernberg O: Inventory of personality organization for adolescents (IPO-A), unpublished)), and has shown satisfactory basic psychometric properties in a non-clinical sample [5].The IPO-A is however yet to be validated in a clinical sample of adolescents. Moreover, as in the majority of previous factor-analytic studies in adults, the initial validation study of the adolescent IPO [5] only examined a simple structure model when rotating the factor loading matrix, excluding alternatives that are equally fitting from a theoretical standpoint. For example, it has been suggested that a bifactor model with a general factor of personality functioning and specific individual factors may be more adequate in the field of personality research [48]. Recent studies investigating a general factor of psychopathology and personality pathology in adults [49–51] also suggest that Kernberg’s model of PO may reflect a general factor of personality functioning.

 The current study therefore aimed to develop and validate a shorter measure of adolescent personality functioning based on Kernberg’s model of PO. For this purpose, the factor structure of the IPO-A was investigated (aim 1) by testing the viability of a bifactor model. We hypothesized that the IPO-A short form (IPO-A-SF) would be composed of a general factor reflecting core features of personality dysfunction, and specific factors (e.g., aggression, moral functioning) that capture stylistic expressions that are independent of overall personality functioning. Subsequently, the convergent and discriminant validity of the IPO-A-SF was examined (aim 2) using biserial correlations with the SCID-5-SPQ diagnoses and external measures of psychological distress. Finally, the clinical utility of the IPO-A-SF was examined (aim 3) to test for the diagnostic accuracy of the IPO-A-SF, as well as its association with the AMPD.

## Method

### Participants and procedure

A total sample of *N* = 525 adolescents aged 13 to 19 years were recruited through a community University-Health Psychology Clinic as current patients (*n* = 94) or who responded to an online research call (*n* = 431). Inclusion criteria were: age between 13–19; currently undergoing psychotherapy and/or have a diagnosed mental health condition. Exclusion criteria were presence of an autistic disorder, or any history of psychotic episodes.

Online participants were recruited through crowd-sourcing platforms (MTurk, Qualtrics) that provided a crowdsource service for a fee, based on the provided selection criteria. A predetermined compensation by the recruitment team was provided to the workers/participants to complete the survey. Online participants were required to pass all attention checks to ensure quality of the data (i.e. minimum completion time of 20 min to screen for speeding, randomly distributed attention checks such as “If you are carefully reading this question, please select the third option below”). Screening measures for personality pathology were included to assign online participants to a PD subgroup (i.e. those meeting criteria for a personality disorder or who have received a diagnosis of personality disorder/report being in therapy for a personality disorder; *n* = 277). A similar process was used for the participants from the Psychology Clinic, using both self-report (BPFS) and clinical-rated (CI-BPD) measures to assign participants to a BPD subgroup (*n* = 34) Participants had a mean age of 15.90 years (*SD* = 3.78) and 71.1% were female. Since online data was collected in two waves (*n*_1_ = 280 from a larger study; *n*_2_ = 151), additional measures were added in the second wave to test for convergent validity and thus were only completed by the second wave participants. See Table 1 for additional information on the subsamples and measures used in this study.

**Table 1:** Overview of samples and instruments used in the study

	Community Psychology Clinic Patients (*N* = 94)					Online Sample (*N* = 431)				
	BPD Patients (*n* = 34)		No BPD Patients (*n* = 60)			PD Patients (*n* = 277)		No PD patients (*n* = 154)		
Measures	*M*	*SD*	*M*	*SD*	Effect size (*d*)	*M*	*SD*	*M*	*SD*	Effect size (*d*)
IPO-A	115.3	19.2	87.5	14.8	1.6	124.4	25.3	91.2	16.8	1.5
BPFSC-11	41.3	4.9	26.5	4.6	3.1	42.5	5.5	27.8	4.6	2.8
CI-BPD	1.3	.8	.4	.7	1.2	-	-	-	-	-
						Online subsample who completed additional measures in second wave (*n* = 151)				
						PD Patients (*n* = 90)		No PD patients (*n* = 61)		
LoPF-Q	-		-			220.6	33.0	179.1	40.0	1.1
PID-5-BF	-		-			35.8	10.2	21.9	11.4	1.3
K-10	-		-			32.7	6.2	23.1	6.4	1.5
SCID-5-SPQ	-		-			3.1	.6	.6	.4	4.9

*IPO-A* Inventory of Personality Organization for Adolescets, *BPFSC-11* Borderline Personality Features Scale for Children, *CI-BPD* Childhood Interview for DSM-IV Borderline Personality Disorder, *LoPF-Q* Levels of Personality Functioning Questionnaire, *PID-5-BF* Personality Inventory for DSM-5 – Brief Form – Child, *K-10* Kessler Psychological Distress Scale, *SCID-5-SPQ* Structured Clinical Interview for DSM-5 Screening Personality Questionnaire

### Measures

#### Inventory of Personality Organization for Adolescents (IPO-A-42; [5])

The IPO-A-42 is a 42-item self-report questionnaire designed to measure the five dimensions of PO in adolescents, namely Identity diffusion (ID), Primitive defenses (PD), Reality testing (RT), Aggression (AG), and Moral functioning (MO). The 42-item version was obtained following the validation of the initial 91-item version (see Biberdzic et al., [5]), and resulted in five empirically derived scales: Stability of Sense of Self and Others (combining the original PD and ID scales; 11 items), Impaired Reality Testing (6 items), Aggression (11 items), Moral Functioning (9 items), and Instability of Goals (5 items from the original ID scale). Items are rated on a 5-point Likert-type scale ranging from never true to always true. The IPO-A has shown sound psychometric properties [3, 36].

#### Levels of Personality Functioning Questionnaire (LoPF-Q; [3])

The LoPF–Q 12–18 [2] is a self-report questionnaire used to assess impaired personality functioning for adolescents from 12 to 18 years old and was inspired by the AMPD. It contains 97 items to be answered on a 5-point scale ranging from 0 (no) to 4 (yes). The resulting four scales—Identity, Self-Direction, Empathy, and Intimacy—are coded toward pathology and add up to a total score ranging from no impairment to severe impairment.

#### Borderline Personality Features Scale for Children-11 (BPFSC-11; [4])

The BPFSC-11 consists of 11 items measuring borderline personality features in childhood (for ages 9 and older, including adolescents). Items in the BPFSC-11 comprise behaviour reflective of core BPD features, namely, affective instability, identity problems, and negative relationships. Sample items include “How I feel about myself changes a lot” and “I want to let some people know how much they’ve hurt me.” These items assess how participants feel about themselves and other people, and are rated on a 5-point Likert-type scale ranging from *not true at all* to *always true*. The BPFSC-11 has shown adequate psychometric properties (Cronbach’s α = 0.85) in a sample of adolescent inpatients [4].

#### Childhood Interview for DSM-IV Borderline Personality Disorder (CI-BPD; [52])

The CI-BPD is a semistructured interview developped to assess BPD in youth. The clinician is asked to rate 9 criteria reflecting BPD features ranging from “0” (no symptoms), “1” (probably present), or “2” (symptoms definitely present). For a full diagnosis of BPD, a minimum of 5 criteria rated “2” are required. In line with the DSM-IV criteria for BPD in adults, the CI-BPD assesses for clinical symptoms of intense anger, chronic feelings of emptiness, affective instability, identity impairment, paranoid features or dissociative symptoms, fear of abandonment, self-harm and suicidality, impulsive behaviour, and persistently chaotic and unstable interpersonal relationships.

#### Kessler psychological distress scale (K-10; [53])

The K-10 was developed as a short screening scale for psychological distress [53]. The scale consists of 10 items on a 5-point scale (1 = none of the time, 5 = all of the time) with total scores ranging from 10 to 50 and higher scores indicating greater levels of psychological distress. Total scores equal to or below 19 indicate mental wellness, scores of 20–24 indicate mild psychological distress, scores of 25–29 indicate moderate distress, and scores equal to or above 30 indicate severe distress.

#### Structured Clinical Interview for DSM-5 Screening Personality Questionnaire (SCID-5-SPQ; [54])

The SCID-5-SPQ is a multiple-choice self-report scale, with 106 items used to screen for the possible presence of personality disorders according to DSM-5 criteria [5]. Only the screening questionnaire was used in this study.

#### Personality Inventory for DSM-5 – Brief Form – Child (PID-5-BF; [7])

The PID-5-BF [5] is a 25-item self-report questionnaire which was designed to assess the five AMPD trait dimensions of Negative Affectivity, Detachment, Antagonism, Disinhibition, and Psychoticism in both adults and adolescents; each domain scale consisting of 5 items. Each PID-5-BF item is scored on only one PID-5-BF trait scale. The PID-5-BF items come from the 220-item self-report PID-5. As in the PID-5, each PID-5-BF item is rated on a 4-point scale (i.e., 0 = very false or often false; 1 = sometimes or somewhat false; 2 = sometimes or somewhat true; 3 = very true or often true).

### Statistical analyses

Confirmatory factor analysis (CFA) and bifactor exploratory structural equation modelling (bifactor-ESEM) were conducted using Mplus version 7.4 [55]. All analyses employed weighted least squares means and variance adjusted (WLSMV) estimation as recommended for handling non-normal ordinal data (Beauducel & Herzberg, 2006). The following approximate fit indexes for model evaluation were used: the Root Mean Square Error of Approximation (RMSEA; adequate fit: < 0.10; good fit: < 0.06), the Comparative Fit Index (CFI; adequate fit: > 0.90; good fit: > 0.95), the Tucker– Lewis Index (TLI; adequate fit: > 0.90; good fit: > 0.95), and the Standardized Root Mean Residual (SRMR; good fit: < 0.08). In line with current recommendations [56–58], we evaluated the bifactor models based on: model fit; whether the general factor is defined by all items; the reliability of the specific factors; and adequate convergent and discriminant validity. Bifactor-ESEM specific fit indices include: omega hierarchical (ωH; good fit: > 0.80), explained common variance (ECV: good fit: > 0.60), and percentage of uncontaminated variance (PUC: > 0.80) and were calculated in r-studio version 4.1 using package Bifactor Indices Calculator version 0.2.2 [59]. In addition to ωH (indicating how much common variance is accounted for by the general factor), additional commonly reported omega coefficients include omega general (ωG; indicating the internal consistency reliability of the general factor when controlling for the effects of all specific factors) and omega specific (ωS; indicating the internal consistency reliability of a specific factor when controlling for the effect of other factors in the model).

To investigate the factor structure of the IPO-A, and to derive a shorter version of the instrument (IPO-A-SF), we first ran a series of exploratory and confirmatory factor analyses. Upon initial inspection, the Instability of Goals scale was found to be problematic with poor factor loadings and weak inter-item correlations. Considering that this issue has been reported in previous studies [31], the Instability of Goals scale was not included in subsequent analyses. We decided to test (a) a single-factor model in which the scales loaded onto a single factor representing the PO dimension; (b) a correlated four-factor model that proposed the previously obtained factors by Biberdzic and colleagues [3] – Stability of Sense of Self and Others, Aggression, Reality Testing, and Moral Functioning; (c) a bifactor CFA model that included a general factor of personality functioning, on which all indicators loaded, and specific factors for the remaining dimensions; and (d) a bifactor ESEM model that also included both a general factor of personality functioning, and specific factors for the remaining dimensions. For CFA models, items were allowed to load onto their a priori factor, and all cross-loadings were constrained to be exactly zero. As for the ESEM model, item loadings were freely estimated. Both the bifactor-CFA model and bifactor-ESEM model were specified to be orthogonal [60]. Through iterations of retaining and deleting items on the basis of their factor loadings and item intercorrelations, the number of items of the IPO-A-SF was reduced to 15.

To investigate the convergent and discriminant validity of the IPO-A-SF we tested whether factor scores were systematically associated with the presence of DSM-5 categorical personality disorders (according to SCID-5-SPQ), external measures of psychological distress (i.e. K-10) and other dimensional measures of personality pathology (e.g. PID-5, LoPF-Q). Lastly, to investigate the clinical utility of the IPO-A-SF, we examined the instrument’s capacity to identify adolescents who present with a personality disorder by comparing it to a clinician-rated interview and gold-standard for assessing BPD in youth (CI-BPD).

## Results

### Establishing a bifactor model

The goodness-of-fit of the four alternative models is reported in Table 2. The single-factor CFA model demonstrated poor fit, indicating the value of looking at the individual dimensions of PO individually. Fit indices for both the four-factor ESEM model and the bifactor CFA were adequate. In contrast, the bifactor-ESEM models for both the IPO-A and the IPO-A-SF provided an excellent degree of fit to the data, resulting in a significant improvement in comparison with the four-factor ESEM model. (∆CFI =  + 0.015 to 0.035; ∆TLI =  + 0.018 to 0.038; ∆SRMR = –0.051 to –0.054).

**Table 2:** Goodness-of-fit statistics for all tested models

	χ^2^	df	RMSEA (90% CI)	CFI	TLI	SRMR
Single-factor CFA	3521.692	405	0.129 (0.125–0.133)	0.655	0.629	0.094
Four-factor ESEM	908.740	325	0.062 (0.058–0.067)	0.935	0.914	0.082
Bifactor CFA	1203.325	384	0.068 (0.064–0.072)	0.909	0.897	0.079
**Bifactor ESEM (IPO-A)**	**771.239**	**321**	**0.055 (0.050–0.060)**	**0.950**	**0.932**	**0.031**
**Bifactor ESEM (IPO-A-SF)**	**282.853**	**116**	**0.056 (0.048–0.064)**	**0.970**	**0.952**	**0.028**

Table 3 presents the loading matrix of the bifactor ESEM model for the IPO-A-SF after orthogonal bifactor rotation. All items showed substantially positive loadings (i.e., > 0.35) on the general factor. Omega hierarchical was ωH = 0.81, indicating high saturation of the common variance with the general factor. Similarly, PUC = 0.85, indicating that the correlation matrix is ‘essentially unidimensional’ [61]. The proportion of common variance explained by the general factor (ECV = 0.51) was however moderate. Nonetheless, PUC moderates the impact of ECV on parameter bias when fitting a unidimensional model (e.g., single-factor model) to multidimensional data (e.g., when the specific factors account for a sizeable proportion of the common variance). In instances where the ECV is low-to-moderate (i.e. the common variance is multidimensional), parameter bias will be less influential if the PUC is high [62]. In oher words, the presence of some multidimensionality in this case is not severe enough to disqualify the strength of the general factor. This suggests that a bifactor model provides a reasonable representation of the latent structure of the IPO-A, as all items were shown to be useful indicators of general impairments in personality functioning. The general factor was saturated with content from all three main theoretical dimensions of PO (i.e., identity diffusion, primitive defenses and reality testing) as well as from the two additional dimensions (i.e., aggression and moral functioning). The three specific factors seemed to capture stylistic expressions of impaired personality functioning in the domains of Aggression, Reality Testing, and Moral Functioning. All three factors had high internal consistency as indicated by their respective omega specific coefficients (ωS = 0.70, 0.51, 0.45), and there was a significant proportion of common variance that was unique to each specific factor (see Table 3).

**Table 3:** Factor loadings from the bifactor ESEM (IPO-A-SF)

Item	Scale	GF	SF1	SF2	SF3
IPO-A7 I feel like my tastes and opinions come from other people	ID	**.57**	-.05	-.10	.03
IPO-A12 I need to admire people in order to feel secure	PD	**.80**	.09	-.03	-.15
IPO-A16 I often have difficulty seeing flaws in people I admire	PD	**.68**	-.17	.08	.01
IPO-A25 When others see me as having succeeded, I’m delighted, but when they see me as failing, I feel devastated	ID	**.43**	-.09	-.17	-.09
IPO-A31 It’s hard for me to be alone	ID	**.45**	-.08	-.13	-.01
IPO-A41 My choices and tastes are influenced by what others say	ID	**.57**	-.09	-.05	.05
IPO-A18 I enjoy hurting others	AG	**.49**	**.74**	.04	-.02
IPO-A26 I find the suffering of other people exciting	AG	**.47**	**.79**	-.01	.02
IPO-A30 I enjoy making other people suffer	AG	**.51**	**.80**	-.02	-.02
IPO-A17 I can see or hear things that others can’t	RT	**.62**	-.01	**.69**	.00
IPO-A19 I hear things that other people claim do not exist	RT	**.64**	-.00	**.68**	-.01
IPO-A21 I have heard or seen things when there is no apparent reason for it	RT	**.62**	.00	**.68**	-.02
IPO-A3 Everybody would steal if they were not afraid of getting caught	MO	**.38**	.03	.01	**.52**
IPO-A11 People pretend feeling guilty when, in fact, they are only afraid of getting caught	MO	**.51**	.04	-.01	**.58**
IPO-A15 One cannot judge others’ real feelings based on their surface behavior because what you see can be manipulated	MO	**.36**	-.09	-.01	**.43**
ω		0.92	0.94	0.94	0.80
ωH		0.81	0.70	0.51	0.45
ECVss		0.51	0.74	0.54	0.64
ECVgs		0.51	0.25	0.46	0.36

*N* = 516; ω = omega coefficient; ωH = omega hierarchical coefficient; ECVss = the proportion of common variance in a subdomain which is unique to that subdomains specific factor; ECVgs = the proportion of common variance of the items in a specific factor explained by the general factor

### Convergent and discriminant validity

Table 4 presents point-biserial correlations of the IPO-A-SF factor scores with SCID-5-SPQ personality disorder diagnoses in a subsample of 151 participants. General personality dysfunction was significantly positively associated with the presence of four PDs, i.e., Dependent, Paranoid, Schizotypal, and Borderline PD. Associations were strongest for Borderline PD, but nonsignificant for Obsessive–Compulsive, Avoidant, Schizoid, Paranoid, Histrionic, Narcissistic, and Antisocial PD. Note that the latter three PDs had the lowest prevalence rates in this sample, which may contribute to this null finding. Aggression was positively associated with the presence of Antisocial PD, and Impairments in Reality Testing showed the strongest association with Schizotypal PD. Finally, impairments in Moral Functioning showed a positive association with Narcissistic and Borderline PD.

**Table 4:** Point-biserial correlations of IPO-A-SF factor scores with SCID-5-SPQ diagnoses

SCID-5-SPQ cut-offs	IPO-A-SF factor scores				*N* (%)
	General personality dysfunction	(High) Aggression	(Impaired) Reality Testing	(Impaired) Moral Functioning	
Obsessive	.179	.196	.169	.072	73 (48.3)
Avoidant	-.003	.040	.081	-.067	79 (52.3)
Dependent	.358**	.161	.048	.183	42 (27.8)
Paranoid	.218*	.119	.105	.173	75 (50.3)
Schizotypal	.299**	-.006	.285**	.173	27 (17.8)
Schizoid	.034	.018	.129	-.026	26 (17.2)
Histrionic	.098	.001	-.031	-.105	14 (9.3)
Narcissistic	-.022	.198	-.109	.214*	28 (18.5)
Borderline	.324**	.080	-.006	.356**	102 (67.5)
Antisocial	.040	.340**	-.108	.128	19 (12.6)

*N* = 151

The number of endorsed PD categories was also positively associated with impairments on all four IPO-A-SF factors, namely General personality dysfunction (*r*(149) = 0.54, *p* < 0.001), Aggression (*r*(149) = 0.39, *p* < 0.001), Reality Testing (*r*(149) = 0.35, *p* < 0.001), and Moral Functioning (*r*(149) = 0.33, *p* < 0.001). There was also a significant difference between those who met criteria for multiple PDs (*M* = 40.96, *SD* = 7.33) and those who met criteria for only one PD (*M* = 33.13, *SD* = 3.87) in terms of General personality dysfunction (*t*(37.63) = 5.98, *p* < 0.001).

Table 5 presents Pearson correlations of the IPO-A-SF factor scores with external measures of psychopathology and functioning. General Personality Dysfunction was significantly positively associated with all external measures of personality pathology. As expected, General Personality Dysfunction was also associated with greater psychological distress.

**Table 5:** Correlations between IPO-A-SF factor scores and external measures of functioning and psychopathology

	IPO-A-SF factor scores			
External measures	General personality dysfunction	Aggression	Reality Testing	Moral Functioning
PID-5-BF				
Negative Affect	.349**	-.025	-.155	.325**
Detachment	.175*	-.109	.142	.130
Antagonism	.363**	.456**	.026	.161
Disinhibition	.364**	.178	.111	.036
Psychoticism	.608**	.105	.461**	.259*
BPFSC Total	.527**	.236**	.190**	.337*
LoPF-Q Total	.642**	.530**	.494**	.394**
Identity	.554**	.218**	.253**	.336**
Self-Direction	.541**	.179**	.275**	.335**
Empathy	.675**	.572**	.271**	.287**
Intimacy	.501**	.241**	.251**	.272**
K-10	.477**	.039	.143	.440**

*N* = 151

*IPO-A* Inventory of Personality Organization for Adolescets, *BPFSC-11* Borderline Personality Features Scale for Children, *CI-BPD* Childhood Interview for DSM-IV Borderline Personality Disorder, *LoPF-Q* Levels of Personality Functioning Questionnaire, *PID-5-BF* Personality Inventory for DSM-5 – Brief Form – Child, *K-10* Kessler Psychological Distress Scale, *SCID-5-SPQ* Structured Clinical Interview for DSM-5 Screening Personality Questionnaire

### Clinical utility

**Fig. 1:**
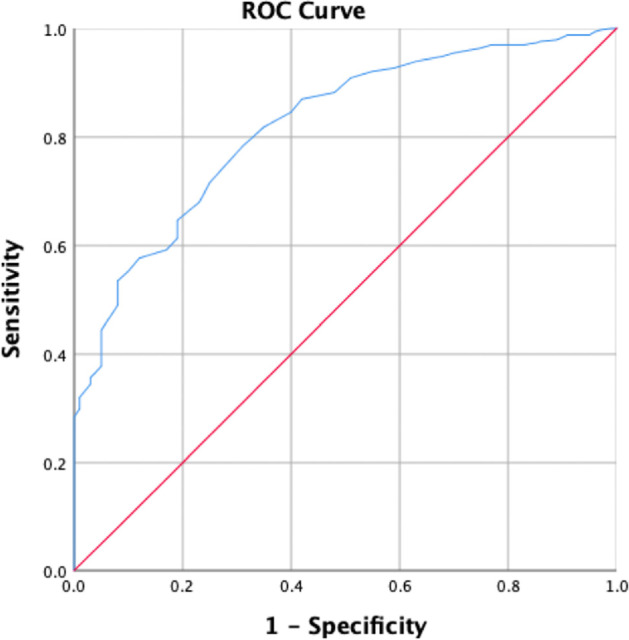
Receiver operating characteristic (ROC) curve for IPO-A-SF’s total score (general factor) in detecting borderline personality pathology as defined by the CI-BPD

Receiver operating characteristic (ROC) analysis was used to further determine the clinical utility of the IPO-A-SF and to derive empirical cutoff scores for defining clinically relevant thresholds (see Fig. 1).

The analysis of adolescents who met criteria for a borderline PD according to the BPFSC-11 cut-offs (*n* = 365) versus adolescents with no relevant signs of borderline personality features (*n* = 159) showed a high predictive power of the IPO-A-SF total score with an area under the curve (AUC) of 0.82, 95% CI [0.78, 0.87], *p* < 0.001 (sensitivity = 0.77; specificity = 0.70). We also examined the diagnostic accuracy of the IPO-A-SF in distinguishing between the BPD adolescent patients (*n* = 34) and the non-BPD ones (*n* = 60). This resulted in an AUC of 0.75, 95% CI [0.64, 0.86], *p* < 0.001 (sensitivity = 0.71; specificity = 0.74). In order to establish cut-off scores, we examined the diagnostic accuracy of the IPO-A-SF using only the clinician-rated adolescent group (*n* = 94) and their scores on the CI-BPD. This revealed an AUC of 0.82, 95% CI [0.67, 0.92], *p* < 0.001, with an optimal cutoff of 32, providing 0.76 sensitivity and 0.82 specificity. For comparative purposes, we also investigated the predictive power of the LoPF-Q in identifying adolescents who met criteria for a borderline PD (using the BPFS-11 cut-offs). The LoPF-Q yielded similar results to the IPO-A-SF when using the self-report measure, with an AUC of 0.82 (sensitivity = 0.70; specificity = 0.82).

We also investigated the predictive power of the individual dimensions of the IPO-A-SF. As expected, only the General Personality Dysfunction factor (the equivalent of the total score) yielded good results in terms of predictive power. These results are reported in Figure S1 of the online supplements.

## Discussion

The broader goal of this study was to develop and validate a shorter measure of adolescent personality functioning based on Kernberg’s model of personality orgnization. Specifically, the aims of this study were threefold: first (1), to empirically test the viability of a bifactor model through CFA and ESEM analyses; second (2), to examine the convergent and discriminant validity of the IPO-A-SF; and lastly (3), to investigate the clinical utility of the IPO-A-SF in detecting impairments in personality functioning in adolescents, in comparison with the AMPD.

### Testing a bifactor model

A bifactor model provided the best fit to the data and consisted of a general factor of personality functioning and three specific factors, representing additional dimensions of PO independent of general personality functioning. A brief 15-item version of the instrument based on the bifactor model was derived, by selecting the items with most significant loadings. Our finding of a general factor of personality pathology in adolescents is consistent with the recent findings by Hörz-Sagstetter and colleagues [63] who found nearly identical results in adults using the IPO. In both studies, many items representing identity diffusion and primitive defenses only loaded on the general factor, without any secondary loadings on specific factors (see Table 2), with the general factor in our study being most highly correlated with the LoPF-Q. This may suggest that the original scales of identity diffusion and primitive defences represent core dimensions of personality functioning, capturing lack of self-other integration similar to the two core indicators described in Criterion A of the AMPD [5]. The identification of a general factor accounting for common variance shared across different personality disorders is also in line with previous work [43, 64]. Specifically, Sharp and colleagues [51] found both general and specific factors of personality pathology in a large sample of adult inpatients, and recently proposed a similar model for understanding personality pathology in adolescents [65]. The fact that the bifactor model (composed of both a general factor and three specific ones) provided the best data fit in this study – and that *all* items loaded on the general factor – also highlight the importance of assessing *both* severity of self- and other-functioning as well as specific domains of psychopathology.

Indeed, the three specific factors established in our bifactor model (i.e. Aggression, Reality Testing, and Moral Functioning) seem to capture stylistic expressions of personality pathology that may provide useful information for differential diagnosis in adolescence. As shown in Table 4, these factors appear to be related to Criterion B in the AMPD [7], with Aggression being most highly correlated with Antagonism; Reality Testing with Psychoticism; and Moral Functioning with Negative Affect. It is however worth mentioning that the inclusion of Reality Testing as a separate, specific factor is divergent from Kernberg’s theory, with reality testing being considered one of the three main dimensions of PO. Interestingly, this was also found to be the case in the recent work by Hörz-Sagstetter and colleagues [63] using the IPO. We agree with the authors’ justification that a frank reality testing impairment is generally not common in individuals with PD, and that it is perhaps most closely linked to schizotypal PD, as was found in both studies.

### Convergent and discriminant validity

With regard to the convergent validity of the factor scores, we found that all four factor scores were significantly associated with the number of endorsed PD categories on the SCID-5-SPQ, and that those who met criteria for multiple PDs had significantly higher scores on all four factors than those who only met criteria for one PD. This suggests, as expected, that impairment on all four domains of the IPO-A-SF is associated with greater levels of severity. Moreover, general personality dysfunction (as measured by the General Factor) was significantly positively associated with the self-reported presence of dependent, paranoid, schizotypal and borderline PD. Associations were however not significant for obsessive–compulsive, avoidant, schizoid, histrionic, narcissistic, and antisocial PD. Some of these null findings may be explained by the very low prevalence rates found in our sample for histrionic, narcissistic and antisocial PDs. Another possible explanation is that some of these clinical presentations are more likely to be captured by the specific factors associated with their stylistic expressions. For example, antisocial PD was uniquely associated with the Aggression factor, while narcissistic PD was significantly linked with impaired Moral Functioning. The latter is consistent with previous findings from Biberdzic and colleagues [3] who found that the moral functioning scale of the IPO captured a narcissistic component that appeared to be most salient in adolescents.

The non-significant association between the specific Aggression factor and borderline personality disorder (BPD) is however surprising as one would expect adolescents with BPD to report high levels of aggression [66]. It is possible that a general impairment in personality functioning (i.e. difficulties in the areas of identity diffusion and primitive defensive functioning) may be a better indicator of BPD in adolescents than aggression. Moreover, the observed non-significant association between BPD and Reality Testing is in line with Kernberg’s model, as BPD is not likely to be associated with general reality testing impairment but only with temporary lapses in reality testing – more often in interpersonal settings and under stress [11]. Finally, as expected, the specific Reality Testing factor was uniquely associated with schizotypal PD, in line with both existing theory and similar empirical research [63].

The differential patterns of the correlations between the bifactor model factors and external measures shown in Table 4 also demonstrate acceptable discriminant validity: the association between General Personality Dysfunction and greater levels of psychological distress is as expected. Similarly, the association between the specific Aggression factor and the PID-5 domain of Antagonism is also expected, as was the correlation between Impaired Reality Testing and the PID-5 domain of Psychoticism. Lastly, the same applies to the positive correlation between the Moral Functioning factor and both the Empathy scale of the LoPF-Q and the BPFSC-11.

### Clinical utility

Finally, the construct validity and clinical utility of the IPO-A-SF were supported, with the total score on the IPO-A-SF successfully discriminating between adolescents who met criteria for borderline personality pathology and those without borderline personality-related difficulties. Indices of specificity and sensitivity as well as the AUC suggested good diagnostic accuracy for the IPO-A-SF when using both the BPFS (self-report) and CI-BPD (clinician-rated) indicators. The predictive power of the IPO-A was also similar to that of the LoPF-Q when compared on the BPFS, while relying on significantly less items. It is also worth highlighting that only the general factor (General Personality Dysfunction) demonstrated high predictive diagnostic value, further supporting the importance of considering all five mentioned domains when assessing personality pathology in adolescence (i.e. identity diffusion, defenses, aggression, reality testing, and moral functioning).

### Alignment with the ICD-11 framework

These findings have important potential implications as they highlight the value of considering other domains as core determinants of personality functioning and severity. Interestingly, despite obvious similarities between the AMPD and ICD-11 frameworks – with the latter also including a possibility to include trait specifiers following the assessment of severity of impairment in self- and interpersonal functioning – it is worth highlighting that the ICD-11 also includes reality testing as a central dimension of PD severity, unlike the AMPD. Furthermore, although aggression towards self and others is assessed in the trait section of the ICD-11, it is noteworthy that the latest ICD-11 screener for PD [67] includes both reality testing and aggression among its core aspects of impairment that are indicative of PD severity [67]. This suggests that the IPO-A-SF is potentially more aligned with the ICD-11 framework, and thus may be of particular use to those who will be using the ICD-11 guidelines for assessing PD in youth.

### Limitations

Nonetheless, certain methodological limitations of the current study should be pointed out. First, the majority of our participants were recruited online through a crowd-sourcing platform. Despite the inclusion of attention checks, the financial incentive may have caused participation bias (e.g. participants endorsing inclusion criteria so they can receive financial compensation). The use of online samples also makes it impossible to confirm the self-reported difficulties. Still, numerous reports have lauded these approaches as capturing high-quality data for research on personality disorders [68], and suggest that participants obtained from crowd-sourcing platforms have also been shown to endorse clinical symptoms to a substantially greater degree than traditional nonclinical samples [69]. Similarly, compensation (and compensation level) has been found to have no impact on data quality [70, 71], thus minimizing the risk of participation bias. Second, convergent validity analyses relied solely on self-report instruments. This is especially challenging when assessing opaque mental structures and processes such as defense mechanisms [72]. The inclusion of clinician-rated measure using a subsample of participants partially addressed this limitation, although the study would have benefited from a larger sample size for this specific subample. Lastly, the number of participants who completed the SCID-5-SPQ is relatively small, which limits the range of personality profiles identified. Considering previous work on the IPO-A using healthy controls, these were not included in the current study. Future studies should however include more heterogeneous samples to cover the full range of psychopathology.

## Conclusion

In conclusion, the results of our study support the use of a bifactor model of personalty organisation in adolescents, in which both general and specific factors are used to assess personality pathology in youth. This is in line with object relations theory and, more specifically, with Kernberg’s approach to diagnosis and classification [22]. Our findings are also compatible with the recently proposed model for the development of personality pathology during adolescence [65] as well as the DSM-5’s AMPD and the recent ICD-11’s classification system [67]. However, we argue that in comparison with the AMPD, the discussed bifactor model relies on a coherent theoretical background (generally missing in the AMPD) that enhances the clinical assessment of personality pathology and provides a road map for treatment planning and intervention (for a detailed comparison between object relations theory and the AMPD, see [20]). The IPO-A also provides the advantage of relying on a single (more integrated and more succinct) measure that captures both the core criterion of self-other functioning as well as other specific domains of functioning that are also core determinants of severity of personality pathology. The successful development of a 15-item version of the IPO-A suggests that the bifactor model can be efficiently implemented in a reduced item set, and may be well-suited for time-efficient screening of personality pathology in youth.

Most importantly, we also argue that the core domains assessed by the IPO-A are more fitting with a developmentally-sensitive approach to personality pathology, and are considerate of the main developmental processes of separation-individuation [73] and identity formation [74] that are crucial in adolescence. Future (longitudinal) studies are however needed to empirically investigate the clinical utility of these domains in youth, and investigate markers of self-other integration over time. Considering that one of the objectives of the current study was to develop a shorter version of the IPO-A, it is possible that an extended version of the instrument may be required to fully capture some of these developmental milestones and other central determinants of personality functioning in adolescence.

## Supplementary Information

**Additional file 1:** **Supplementary Figure 1.** Receiver operating characteristic (ROC) curves for the IPO-A-SF’s specific factors (Aggression, Reality Testing, and Moral Functioning) in detecting borderline personality pathology as defined by the CI-BPD. AUCs: 0.54, 0.56, 0.76.

## Data Availability

The datasets generated and/or analysed during the current study do not have clearance to be made publicly available but the analysis script can be made obtained from the corresponding author on reasonable request.

## Abbreviations

PD: Personality Disorder
IDC-11: International Classification of Diseases
AMPD: Alternative Model of Personality Disorders
IPO-A: Inventory of Personality Organization for Adolescents
LPFS: Level of Personality Functioning Scale
PID-5: Personality Inventory for DSM-5
PO: Personality organization
LoPF-Q: Levels of Personality Functioning Questionnaire for adolescents
STIPO: Structured Interview of Personality Organisation
IPO: Inventory of Personality Organization
IPO-A-SF: Inventory of Personality Organization for Adolescents—Short Form
SSO: Stability of Sense of Self and Others
RT: Impaired Reality Testing
AGG: Aggression
MOR: Moral Functioning
IG: Instability of Goals
BPFSC-11: Borderline Personality Features Scale for Children-11
CI-BPD: Childhood Interview for DSM-IV Borderline Personality Disorder
K-10: Kessler Psychological Distress Scale
SCID-5-SPQ: Structured Clinical Interview for DSM-5 Screening Personality Questionnaire
PID-5-BF: Personality Inventory for DSM-5 – Brief Form – Child
NA: Negative Affectivity
De: Detachment
An: Antagonism
Di: Disinhibition
Ps: Psychoticism
CFA: Confirmatory factor analysis
bifactor-ESEM: Bifactor Exploratory Structual Equation Modelling
WLSMV: Weighted least squares means and variance adjusted
RMSEA: Root Mean Square Error of Approximation
CFI: Comparative Fit Index
TLI: Tucker– Lewis Index
SRMR: Standardized Root Mean Residual
ωH: Omega hierarchical
ECV: Explained common variance
PUC: Percentage of uncontaminated variance
ωG: Omega general
ωS: Omega specific
ROC: Reciever operating characteristic
AUC: Area under the curve
BPD: Borderline personality disorder
