# Supplementary material for: A bifactor model of personality organization in adolescence: the validity of a brief screening measure assessing severity and core domains of functioning
Source: BMC Psychiatry. 2022 Jul 8;22:459. doi: 10.1186/s12888-022-03926-y (PMC9270814; doi:10.1186/s12888-022-03926-y)
Supplement: Supplementary file 1 — Additional file 1: Supplementary Figure 1. Receiver operating characteristic (ROC) curves for the IPO-A-SF’s specific factors (Aggression, Reality Testing, and Moral Functioning) in detecting borderline personality pathology as defined by the CI-BPD. AUCs: 0.54, 0.56, 0.76. [file 12888_2022_3926_MOESM1_ESM.docx]

SUPPLEMENTARY MATERIAL


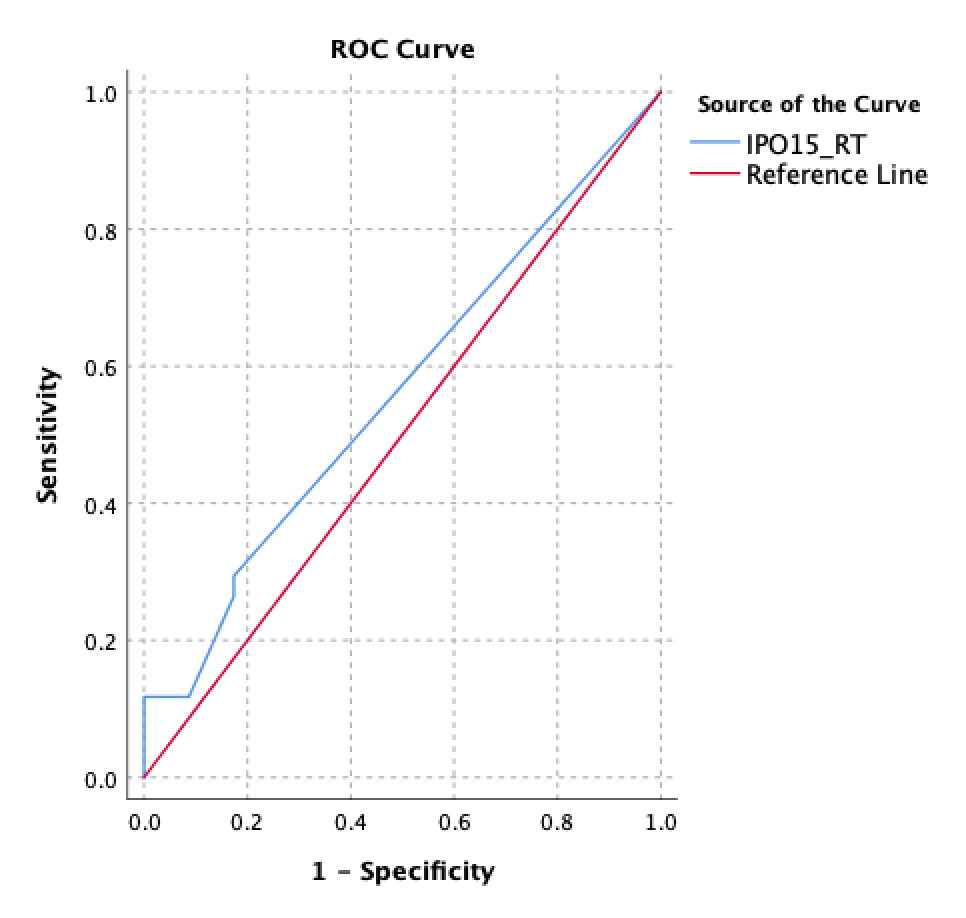

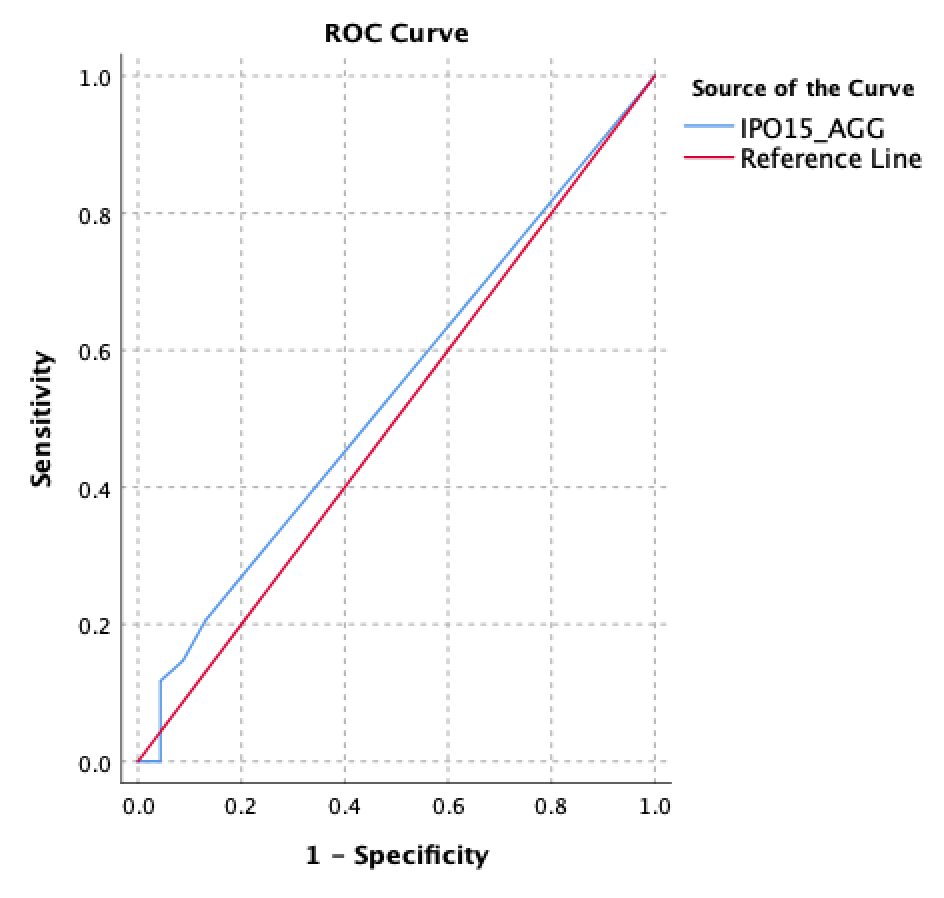


*
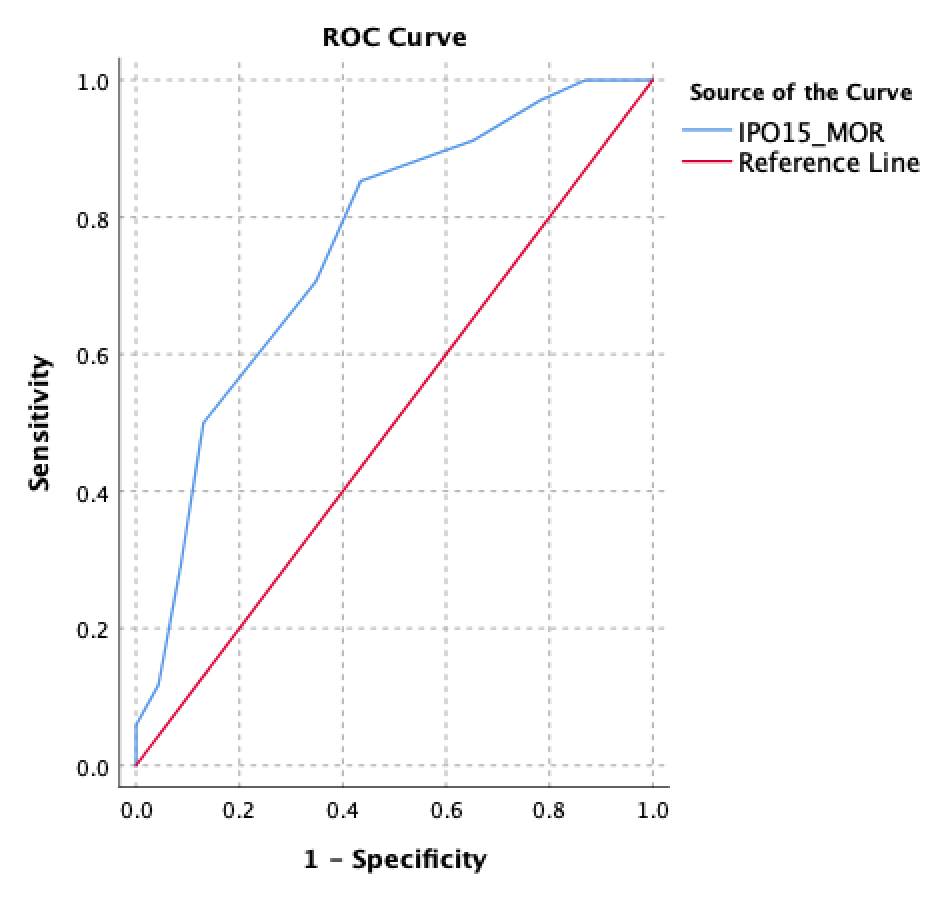
*

*Supplementary Figure 1.* Receiver operating characteristic (ROC) curves for the IPO-A-SF’s specific factors (Aggression, Reality Testing, and Moral Functioning) in detecting borderline personality pathology as defined by the CI-BPD. AUCs: 0.54, 0.56, 0.76
